# Increase in Legionnaires’ disease cases associated with travel to Dubai among travellers from the United Kingdom, Sweden and the Netherlands, October 2016 to end August 2017

**DOI:** 10.2807/1560-7917.ES.2017.22.38.30618

**Published:** 2017-09-21

**Authors:** Gavin Dabrera, Petra Brandsema, Margareta Lofdahl, Falguni Naik, Ross Cameron, Jim McMenamin, Richard Pebody, Nick Phin

**Affiliations:** 1 Centre for Infectious Disease Surveillance and Control, National Infection Service, Public Health England, London, United Kingdom; 2 Epidemiology and Surveillance of infectious diseases, Centre for Infectious Disease Control Netherlands, National Institute for Public health and the Environment (RIVM), Bilthoven, The Netherlands; 3 Surveillance and Coordination, Folkhalsomyndigheten, Stockholm, Sweden; 4 Health Protection Scotland, NHS National Services Scotland, Glasgow, United Kingdom

**Keywords:** Legionnaires' disease, Legionella, outbreaks, travel, epidemiology

## Abstract

Between 1 October 2016 and 31 August 2017, 51 Legionnaires’ disease (LD) cases from the United Kingdom, Sweden and the Netherlands were identified with associated travel to Dubai. Cases did not all stay in the same accommodation, indicating that no single accommodation could be the source for all these infections. While local investigations continue into other potential sources, clinicians should remain alert to the possibility of LD among travellers returning from Dubai with respiratory illness.

In December 2016, the European Centre for Disease Prevention and Control (ECDC) reported an increase in Legionnaires’ disease (LD) cases associated with travel to Dubai, United Arab Emirates (UAE) [1] based on cases reported to ELDSNet (European Legionnaires’ disease surveillance network), an ECDC–operated surveillance system among European Union (EU) countries, Iceland and Norway [2] for laboratory-confirmed, travel-associated LD (TALD) cases who stayed in commercial accommodation site(s) (e.g. hotels) during the 2–10-day incubation period. As this increase in Dubai-associated TALD cases continues, we describe cases reported with symptom onset between 1 October 2016 and 31 August 2017 among residents from the United Kingdom (UK), Sweden and the Netherlands (the three countries that were initially reporting the largest numbers of cases). We describe the ongoing situation as at 18 September 2017 to provide further insight into the observed increase and create awareness among physicians and travellers returning with compatible symptoms to consider legionella as a differential diagnosis [1].

## Case definition

Cases were classified as any person resident in the UK, Sweden or the Netherlands with clinical or radiological evidence of pneumonia, laboratory confirmation of Legionella infection and stay in Dubai during the 2–10 days before symptom onset. Laboratory confirmation included methods fulfilling the EU confirmed case definition (culture testing, urine antigen testing or serology) or the detection of *Legionella* spp. by PCR [3]. All patients meeting the case definition with symptom onset between 1 October 2016 and 31 August 2017, were described by age, sex, tobacco smoking status, presence of underlying medical conditions, laboratory confirmation method and where available, sequence-based typing.

## Surveillance

The LD surveillance systems in our countries have been described elsewhere [4-7]. In brief, LD must be notified by medical practitioners to public health authorities, who interview the patient or close family members to identify potential legionella exposures during the incubation period, including any travel away from the patient’s main residence (national and/or foreign travel).

## Description of cases

**Figure 1:**
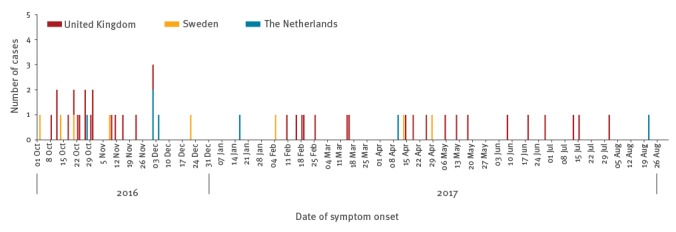
Cases of Legionnaires’ disease associated with travel to Dubai in residents of the United Kingdom, Sweden and the Netherlands, 1 October 2016−31 August 2017 (n=51)

There were 51 laboratory-confirmed LD cases associated with travel to Dubai with symptom onset between 1 October 2016 and 31 August 2017. Of these, 36 were reported from the UK, eight from Sweden and seven from the Netherlands. Over two thirds of cases were men (36/51). The median age for all cases was 66 years (interquartile range (IQR): 57.5−72.0; range: 36−84 years). For cases with available information, 23/43 were smokers or ex-smokers and 18/44 had at least one long-term underlying medical condition. Just over a quarter of cases with hospitalisation information (10/36), required critical care admission. Figure 1 shows the distribution of cases by symptom onset date, and indicates cases concentrated in October 2016, with continuing elevated numbers in November, early December 2016, February and April 2017.

The most frequently used diagnostic method was urinary antigen testing (45/51 cases) with PCR methods used in 17/51 cases and culture in 7/51 cases; 14 cases were diagnosed using more than one type of laboratory test. Most cases were speciated as *Legionella pneumophila* (50/51), while the remaining case was *Legionella* spp. unknown. Serogroup was known for 16 cases: 13 were serogroup 1, two serogroup 13 and one serogroup 2–14. Full sequence-based typing (ST) was available for nine cases (6 cases ST616, 3 cases ST1327) and three cases had partial ST profiles.

The median number of days within the incubation period that cases spent in Dubai was six (IQR: 4−8, range 2–9 days). Eleven cases spent the whole incubation period in Dubai. Four cases also travelled to other emirates within UAE; six had foreign travel to four different countries in addition to UAE, during the incubation period.

In relation to potential aerosol exposures in Dubai, 44 of 48 cases with available information had used showers, while 29 of 45 recalled exposures to fountains and water features. Use of swimming pools (12/40) and spa pools (5/39) was less frequent among cases. Other activities for cases included boat trips (7 cases), fountain shows and visits to the gold souk (3 cases each).

The 51 cases were associated with 45 individual accommodation sites. Most (43/51) cases stayed in commercial accommodation: 27/43 in single accommodation (sites where there were no other associated cases in the previous two years), 15/43 in cluster accommodation (sites where there were two or more associated cases in a two-year period) and 1/43 in a commercial accommodation with limited information (preventing the identification of associated cases in the previous two years); seven stayed in private accommodation and for one case the accommodation type was unknown. Significantly, in addition to the seven private accommodation sites, there were 27 different commercial accommodation sites not previously associated with LD cases (i.e. not part of clusters themselves). Three of the 51 cases were associated with a foreign-travel related cluster in other countries. The cases for whom sequence-based typing was available were all linked to different accommodations sites. The average case numbers recorded for each month by onset date between October and August for 2013−14, 2014−15, 2015−16 and - separately for October 2016 to August 2017 for our three countries, are summarised in the Table, which shows that during the investigation period, the number of Dubai-associated TALD cases was higher than the rounded average case numbers of 3−12 cases for the equivalent 11-month period for each of our countries between 2013 and 2016.

**Table:** Average numbers of Legionnaires’ disease cases associated with travel to Dubai between 1 October and 31 August for 2013−14, 2014−15 and 2015−16 compared with case numbers for 2016−17

Country of residence	Number of LD cases associated with travel to Dubai (1 Oct 2016−31 Aug 17)	Rounded average number of LD cases associated with travel to Dubai (1 Oct 2013−15 to 31 Aug 2014−16)
United Kingdom	36	12
Sweden	8	3
The Netherlands	7	4

LD: Legionnaires’ disease.

**Figure 2:**
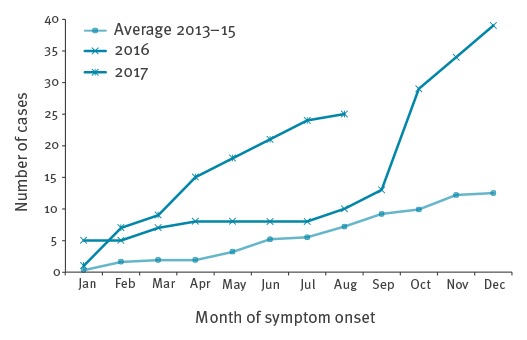
Cumulative number of Legionnaires’ disease cases associated with travel to Dubai in residents of the United Kingdom, Sweden and the Netherlands, January 2016−August 2017 compared to the average for 2013−15

Figure 2 shows that cumulative case numbers for all three countries were particularly elevated from September 2016 onwards, compared with average monthly cumulative case numbers reported for 2013–15. This was sustained for all three countries through to August 2017.

Cumulative numbers for year to date, shown for 2016 and January–August 2017, with average cumulative numbers for 2013–15 for United Kingdom, Sweden and the Netherlands, combined.

## Discussion

Here we report an increase in Dubai-associated TALD cases, above the historical averages for each of our three countries. As our countries’ travellers accounted for the majority of the increase when first observed by ECDC [1], they provide an important insight into this situation.

The hypothesis of increased LD risk related to travel to Dubai rather than increased risk in the countries of residence is supported by the simultaneous peak in cases occurring from September 2016 onwards for all three countries (Table). Additionally, there were no changes in LD surveillance and no community outbreaks in our countries that provide an alternative explanation. Six cases were typed as ST616, a strain observed in our surveillance only in cases associated with travel to Dubai. In addition, ST1327 has been associated with travel to Dubai for all but one case in our surveillance (data not shown). Furthermore, 11 cases spent their entire incubation period in Dubai. These findings support the assertion that these infections occurred within Dubai.

There are several potential explanations for the observed increase in cases associated with travel to Dubai; one could be changes in patient-level risk factors for infection. However, this is unlikely to explain the increase as patient-level risk factors such as underlying medical conditions, current or previous smoking status were reported for 36 cases, which is consistent with almost three quarters among LD cases, previously reported in England and Wales in 2015 [7].

Another potential explanation is an increase in travellers, although overall changes in traveller numbers do not solely explain the scale of the observed increase. UK traveller numbers to Dubai increased by 5% for the calendar year from 2015 to 2016 [8] while travellers to UAE from the Netherlands increased by 9% between November 2016 and April 2017 compared with the same time period one year earlier (data not shown); however, the proportion of the latter dataset related to Dubai specifically is unknown. Due to the incomplete availability of Dubai-specific travel data for all three countries as well as the limited comparability of these available data sources, we have not specifically estimated an incidence rate for LD cases associated with travel to Dubai.

Another explanation is one or more new legionella source(s) in Dubai, although there is limited information available from local investigations. TALD investigations generally focus on accommodation sites [2]. However, these accommodation sites seem an unlikely explanation as only 15 cases were associated with cluster sites. Therefore, wider environmental investigations in addition to commercial accommodation sites are required to identify and control the source of infection.

ECDC reported that there has been no increase in pneumonia notifications locally in Dubai between October and December 2016 [9]. This could be explained by an environmental source where foreign travellers are more frequently exposed than local residents. Alternatively, the local Dubai population is predominantly young, with only 8.7% in the ≥50 age group in 2016, and therefore is potentially at lower LD risk [10].

Several public health actions have been undertaken. To support local investigations, anonymised epidemiological information collected by the public health agencies in the EU countries has been shared with public health authorities in Dubai by ECDC and the World Health Organization (WHO). This supplements our routine international TALD case reporting via ELDSNet. Similar information sharing for 17 TALD cases from different countries was successful in controlling an outbreak in Lazise, Italy, in 2011 [11].

Although the absolute numbers of Dubai-related TALD cases are low in comparison to annual visitor numbers, information for travellers about LD symptoms to be aware of has been published on websites of national public health institutes and on websites for travel health [12-14].

This investigation highlights the benefits of international TALD surveillance (such as ELDSNet) in facilitating identification and alerting of increases in cases related to specific destinations; in addition, ascertainment of cases linked to private accommodation, as well as commercial accommodation, through such surveillance, would be important to determine the full scale of TALD in this particular situation. It should be noted that there would be an inherent delay in reporting to ECDC due to the time taken for diagnosis and collation of relevant epidemiological data.

Seasonal trends in travel to Dubai highlight the potential for further cases occurring in the coming months; for instance travel from the UK is particularly high during the winter and spring seasons [8]. Thus further environmental investigations remain important. While local public health authorities in Dubai conduct public health investigations and implement controls, clinicians should remain vigilant for LD symptoms among returning travellers, to aid prompt diagnosis and treatment.
